# IL-21/IL-21R signaling suppresses intestinal inflammation induced by DSS through regulation of Th responses in lamina propria in mice

**DOI:** 10.1038/srep31881

**Published:** 2016-08-22

**Authors:** Yuanyuan Wang, Xuefeng Jiang, Junfeng Zhu, Dan Yue, Xiaoqing Zhang, Xiao Wang, Yong You, Biao Wang, Ying Xu, Changlong Lu, Xun Sun, Yasunobu Yoshikai

**Affiliations:** 1 Department of anesthesiology, The Fourth Affiliated Hospital, China Medical University, Shenyang, China; 2 Department of Immunology, China Medical University, Shenyang, China; 3 Life Science School, Liaoning University, Shenyang, China; 4 Laboratory Medicine Department, Sheng Jing Hospital of China Medical University, Shenyang, China; 5 Department of Biochemistry and Molecular Biology, College of Basic Medical Sciences of China Medical University, Shenyang, China; 6 Northeast Pharmaceutical Group Co., Ltd, Shenyang, China; 7 Division of Host Defense, Center for Prevention of Infectious Disease, Medical Institute of Bioregulation, Kyushu University, Fukuoka, Japan

## Abstract

Serum level of IL-21 is increased in patients with inflammatory bowel diseases (IBD), suggesting that IL-21/IL-21 receptor (IL-21R) signaling may be involved in the pathogenesis of IBD. However, the role of IL-21/IL-21 receptor signaling plays in the pathogenesis of IBD is not very clear. In this study, using IL-21R.KO mice, we tested the role of IL-21/IL-21R signaling in the regulation of T helper cell responses during intestinal inflammation. Here we found that IL-21R.KO mice were more susceptible to DSS-induced colitis as compared with C57BL/6 mice. The spontaneous inflammatory cytokines released by macrophages in LP of colon were significantly increased, and Th2, Th17 and Treg responses were down-regulated markedly. However, Th1 responses were significantly up-regulated in IL-21R.KO mice. Meanwhile, the population of CD8^+^CD44^+^IFN-γ^+^ T cells was markedly elevated in LP of inflammatory intestine of IL-21RKO mice. *In vivo,* after disease onset, DSS-induced intestinal inflammation was ameliorated in C57BL/6 mice treated with rIL-21. Our results demonstrate that IL-21/IL-21R signaling contributes to protection against DSS-induced acute colitis through suppression of Th1 and activation of Th2, Th17 and Treg responses in mice. Therefore, therapeutic manipulation of IL-21/IL-21R activity may allow improved immunotherapy for IBD and other inflammatory diseases associated with Th cell responses.

Ulcerative colitis (UC) and Crohn’s disease (CD) are inflammatory bowel diseases (IBD) characterized by the activated mucosal immune system and impaired epithelial barrier function and tissue destruction^1^. Although the precise etiology of IBDs remains unclear, imbalanced cytokine production and T cell dysfunction are considered as the key causes for IBD pathogenesis^2^. It has been reported that CD is associated with the cytokines released by T-helper (Th) 1 or Th17^3^,^4^. And UC is related with the releasement of some Th2-type cytokines, such as interleukins (IL) 4, 5, and 13^5^,^6^.

IL-21 is one of the newest member of the common γ-chain cytokine family. including IL-2, IL-4, IL-7, IL-9, IL-13, and IL-15^7^,^8^,^9^. IL-21 is mainly derived from T follicular helper (TFH) and Th17 cells, although it can be produced by some subsets of CD4^+^ T cells^10^,^11^. In addition, it can also be produced by natural killer T (NKT) cells and neutrophils^12^,^13^. Composed of IL-21R α-chain and the common γ-chain subunits^7^,^8^,^14^. IL-21 receptor (IL-21R) is expressed predominantly in immune cells including T cells, B cells, natural killer (NK) cells, macrophages and dendritic cell (DC)^8^,^9^,^15^, which is necessary for intracellular signals transduction. Binding of IL-21 to the IL-21R α/γ chain complex activates the JAK1 and JAK3 pathways which then activates STAT3, as well as STAT1, STAT5a and STAT5b to a lesser extent^7^,^16^. In addition to the STAT pathways, IL-21 triggers the activation of phosphoinositide 3-kinase (PI3K)/Akt and MAP kinase pathways, which are both necessary for IL-21 mediated cell proliferation^17^.

There are several lines of evidence showing that IL-21/IL-21R signaling plays a clear role in promotion of the Th2 cell differentiation and Th2-association cytokines production that specifically inhibits the differentiation of naive Th cells into IFN-γ-producing Th1 cells, *in vivo* and *in vitro*^18^,^19^,^20^,^21^. On the contrary, a recent study shows that IL-21 suppresses the development of Th2 cells and the functions of polarized Th2 cells in in mice with allergic diseases^22^. Meanwhile, it is reported that IL-21 enhances T-helper cell type I signaling and IFN-γ production in CD^23^. It has been shown that the production of IL-17A was reduced in IL-21 KO mice exposed to DSS, compared with that in wild-type mice^24^. IL-21 inhibited the viability of Treg cells by inhibiting IL-2 production^25^, and counteracted Treg suppression by co-stimulating CD4^+^ T cell proliferation and IFN-γ production in response to TCR stimulation^26^,^27^. Numerous studies have demonstrated that IL-21 can significantly enhance the proliferation, survival and cytotoxic functions of CD8^+^ T lymphocytes^28^. In contrast, it was shown that stimulation of antigen-activated CD8^+^ T cells with IL-21 results in decreased cytotoxicity and lower expression of CD44 and IFN-γ^29^. Hence, the roles of IL-21/IL-21R in regulations of Th cell differentiation and functions are very intricate and not clear, at least for now.

Recent studies indicated that the amount of IL-21 was significantly increased in peripheral blood and intestinal tissue of patients with CD or UC^23^,^30^, suggesting that IL21/IL21R signaling is involved in the pathogenesis of IBD. However, little is known about the roles of IL21/IL21R signaling in IBD. Therefore, we conducted following work to try to provide evidences to elucidate the roles of IL21/IL21R signaling in regulation of the mucosa immune responses during intestinal inflammation.

## Results

### IL-21RKO Mice are Susceptible to DSS-induced Intestinal Inflammation in Mice

**Figure 1:**
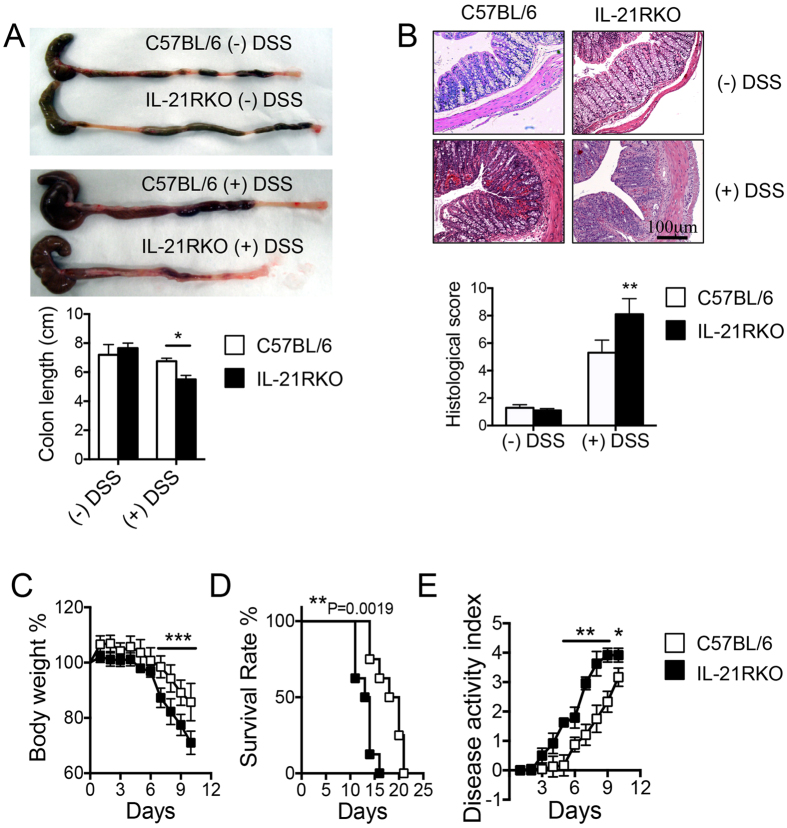
Susceptibility of IL-21RKO mice to DSS-induced colitis. Mice were oral treated with 3% DSS in the drinking water to induce colitis as described in Methods. On day 5, (**A**) macroscopic changes, colon length and (**B**) histological score (original magnification, ×200) were analyzed. (**C**) body weight, (**D**) survival rate and (**E**) disease activity index were daily observed. Data indicate mean ± SD of 10~15 mice of each group obtained from a representative of three independent experiments. Statistically significant differences from the value for DSS-treated C57BL/6 mice are shown (*P < 0.05, **P < 0.01 or ***P < 0.001).

We firstly examined the susceptibility of IL-21R deficient mice to DSS-induced colitis. As shown in the Fig. 1A, macroscopic examinations on day 5 after 3% DSS administration revealed that the colon was markedly shorter in IL-21RKO mice than that in C57BL/6 mice (*P < 0.05). On histological examination, crypt damage, ulceration, and infiltration of inflammatory cells were significantly aggravated in the colons of DSS-treated IL-21RKO mice as compared with C57BL/6 mice and histological analysis of colons from IL-21RKO mice showed greatly increased numbers of infiltrating cells, degree of mucosal injury and edema (Fig. 1B). The histological score of the colons was significantly higher in IL-21RKO mice than that in C57BL/6 mice after DSS administration (Fig. 1B, **P < 0.01). Comparison with C57BL/6 mice, IL-21RKO mice showed exacerbated intestinal inflammation as indicated by the significant weight loss from day 7 to day 10 (Fig. 1C, ***P < 0.001) and the survival rates significantly decreased after 3% DSS administration (Fig. 1D, **P = 0.0019). Meanwhile, we found that DAI score significantly increased from day 6 to day 10 in IL-21RKO mice as compared to that in C57BL/6 mice after DSS-treatment (Fig. 1E, *P < 0.05; **P < 0.01). All of these results demonstrated that IL-21R deficiency results in increased susceptibility to DSS-induced intestinal inflammation in mice.

### Cell Accumulation in LP of Inflammatory Intestine in Mice

**Figure 2:**
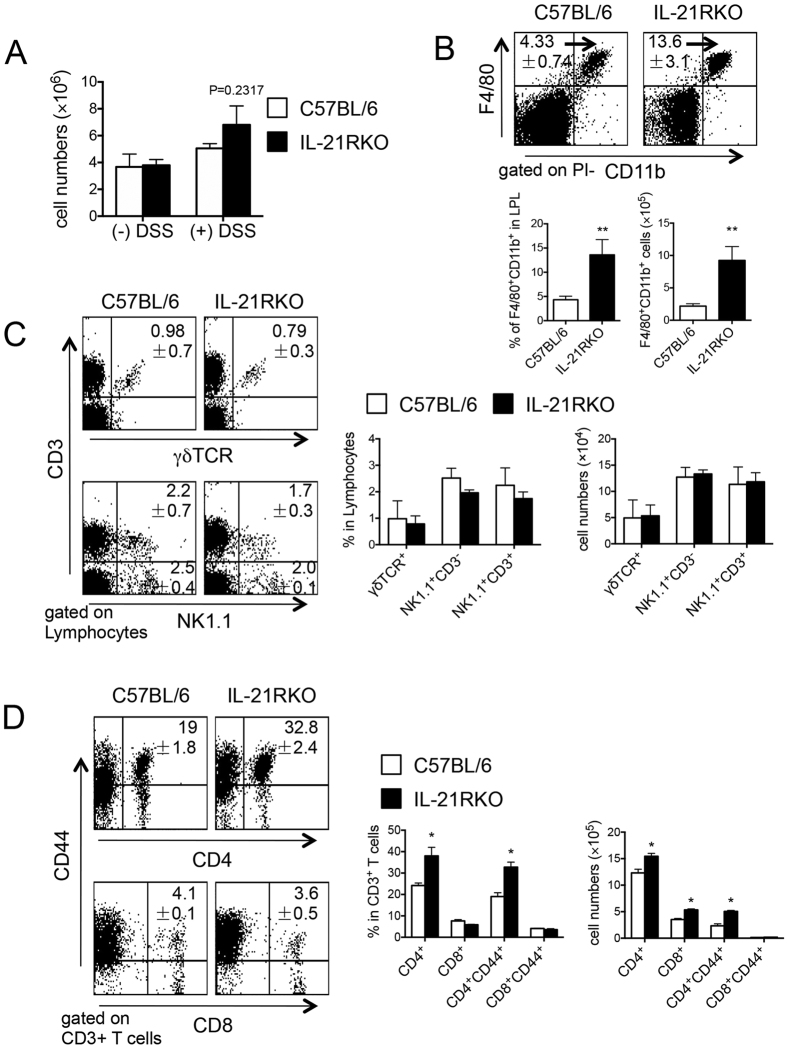
Flow cytometry analysis of the populations of LPL in the colon of mice with DSS-induced colitis. LPL were isolated from inflammatory intestinal tissues of mice on day 5 after DSS-induced colitis and measured by flow cytometry. (**A**) The total number of LPL in the colon in IL-21RKO and C57BL/6 mice on day 5 after DSS-induced colitis. The proportion and absolute number of (**B**) macrophage (F4/80^+^CD11b^+^), (**C**) NK (NK1.1^+^CD3^−^), γδ T (γδTCR^+^CD3^+^) or NKT (NK1.1^+^CD3^+^) cell and (**D**) CD4^+^, CD8^+^, CD4^+^CD44^+^, CD8^+^CD44^+^ T cells. Data indicate mean ± SD of 5 mice of each group obtained from a representative of three independent experiments. Statistically significant differences are shown (*P < 0.05).

Populations of LPL in the colon from IL-21RKO mice and C57BL/6 mice on day 5 after 3% DSS administration were analyzed by flow cytometry. The absolute numbers of total LPL of colon increased after DSS-treatment in both C57BL/6 and IL-21RKO mice, but there is no difference between the two (Fig. 2A). And then we examined the infiltration of macrophage (F4/80^+^CD11b^+^), as shown in Fig. 2B, both the percentage and absolute number of macrophages significantly increased in the inflammatory intestinal tissues of IL-21RKO mice as compared with those in C57BL/6 mice (*P < 0.05). However, it is no markedly change in the accumulation of γδT cell (γδTCR^+^CD3^+^), NK (NK1.1^+^CD3^−^) or NKT (NK1.1^+^CD3^+^) cell in LP of colon between C57BL/6 and IL-21RKO mice (Fig. 2C). We also examined the populations of T cell subsets in the LP, it was found that the proportions and absolute numbers of CD4^+^ and CD4^+^CD44^+^ T cells were significantly higher in DSS-treated IL-21RKO mice than those in C57BL/6 mice (*P < 0.05), whereas the cell number, but not percentage of CD8^+^ T cells had distinctly increased in DSS-treated IL-21RKO mice as compared with C57BL/6 mice (Fig. 2D, *P < 0.05).

### Production of Spontaneous Inflammatory Cytokine in DSS-Treated Intestinal Tissues

**Figure 3:**
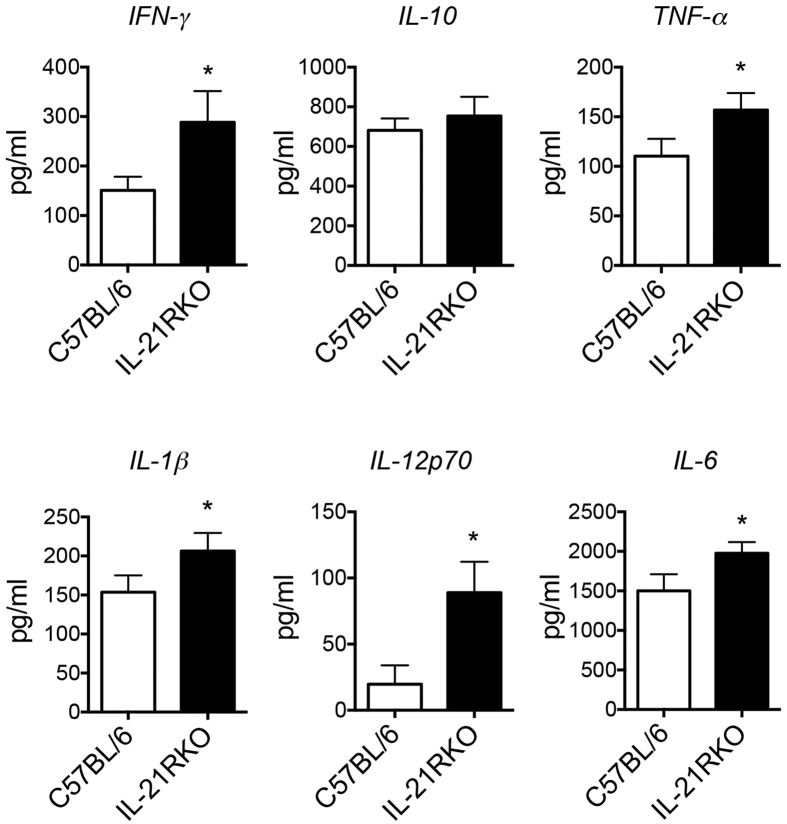
Spontaneous inflammatory cytokine production by LP cells in mice with DSS-inducted colitis. LPL from colon of DSS-treated IL-21RKO or C57BL/6 mice was isolated and cultured for 24 h without any stimulation. The level of cytokine production in culture supernatant was measured using ELISA kits. Data indicate mean ± SD of 8 mice of each group obtained from a representative of three independent experiments. Statistically significant differences from the value for DSS-treated C57BL/6 mice are shown (*P < 0.05).

Many of monocytes, such as macrophages, infiltrated markedly to the inflammatory intestinal tissues in IL-21RKO mice after DSS administration (Fig. 2B). These infiltrated cells are contributing factors for induction of the innate and adaptive immune responses in mucosa through producing various pro-inflammatory cytokines. So we next measured the spontaneous cytokines secreted by lamina propria (LP) cells in colon of DSS-treated mice. As shown in the Fig. 3, the levels of IFN-γ, TNF-γ, IL-1β, IL-12p70 and IL-6, but not IL-10, were significantly higher in IL-21RKO mice than those in C57BL/6 mice on day 5 after DSS-induced colitis (*P < 0.05). Therefore, these results suggest that IL-21R deficiency results in increased infiltration of macrophages and secretion high levels of pro-inflammatory cytokines in LP of inflammatory intestinal tissue in mice.

### T cell-mediated immune responses in LP of mice during DSS-induced intestinal inflammatory

**Figure 4:**
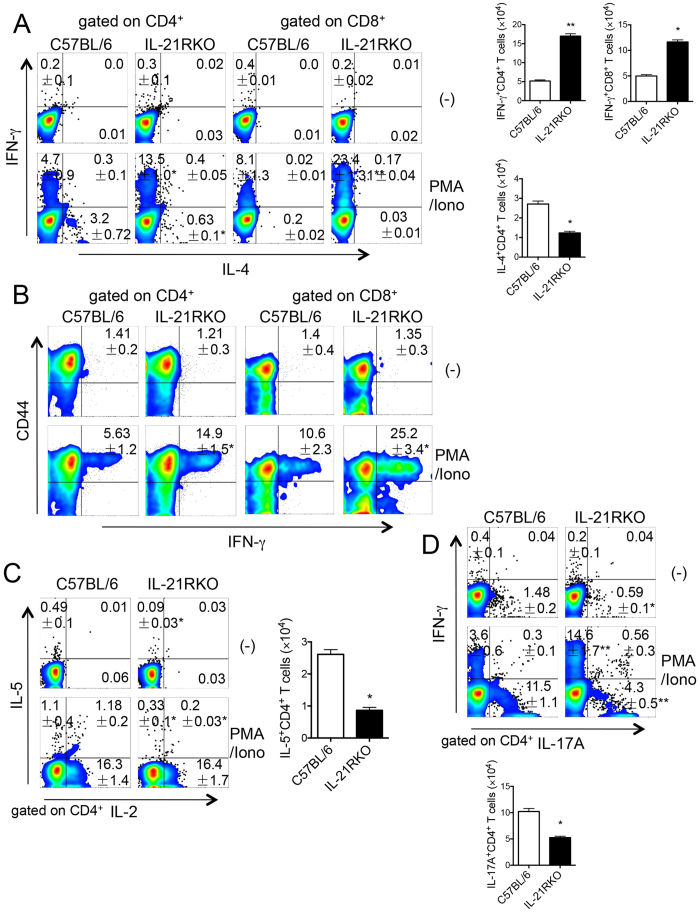
Cytokine-producing T cells in the LPL of colon in IL-21RKO mice with DSS-induced colitis. (**A**) The proportions and absolute numbers of IFN-γ^+^CD4^+^, IFN-γ^+^CD8^+^, IL-4^+^CD4^+^ T in LPL of colon; (**B**) The percentage of CD44^+^IFN-γ^+^ in CD4^+^ or CD8^+^ T cells in LPL of colon; (**C**) The proportion and absolute number of IL-5^+^CD4^+^ T cells in LPL of colon; (**D**) The proportion and absolute number of IL-17A^+^CD4^+^ T cells in LPL of colon. Data indicate mean ± SD of 3 mice of each group obtained from a representative of three independent experiments. Statistically significant differences are shown (*P < 0.05 or **P < 0.01).

As we know, effector T helper cell (Th), such as Th1, Th2 and Th17, and the associated cytokines-mediated immune responses are very importance in the development of intestinal inflammation not only in mouse colitis models but also in patients with IBD. Moreover, many studies had been reported that IL-21/IL-21R signaling plays an important role in the regulation of T cell differentiations *in vivo* and *in vitro*^10^,^22^,^31^. Therefore, we next examined the T cell differentiations in LP of the inflammatory colonic tissues of mice on day 5 after DSS-treatment. As shown in Fig. 4A, the percentage and cell number of Th1 cells, bearing INF-γ-producing CD4^+^ T cells, significantly elevated in LP of IL-21RKO mice as compared to C57BL/6 mice (*P < 0.05; **P < 0.01). The expression of IFN-γ in CD8^+^ T cells was also greatly higher in IL-21RKO mice than that in C57BL/6 mice (Fig. 4A, **P < 0.01; *P < 0.05). Moreover, almost IFN-γ-producing CD4^+^ or CD8^+^ T cells expressed CD44, a marker of effector/memory T cell, and the percentage of IFN-γ^+^CD44^+^ in CD4^+^ or CD8^+^ T cells was significantly upregulated in IL-21RKO mice as compared with that in C57BL/6 mice (Fig. 4B, *P < 0.05). However, Th2 and Th17 differentiations were markedly suppressed displayed significant decrease expression of in IL-4, IL-5 or IL-17A in CD4^+^ T cells in IL-21RKO mice as compared to those in C57BL/6 mice (Fig. 4A,C,D, *P < 0.05). These results suggest that IL-21/IL-21R signaling has an ability to regulate Th1, Th2 and Th17 differentiations in LP during DSS-induced intestinal inflammation.

### Th Cell-Associated Cytokines Production in LP of Inflammatory Intestine

**Figure 5:**
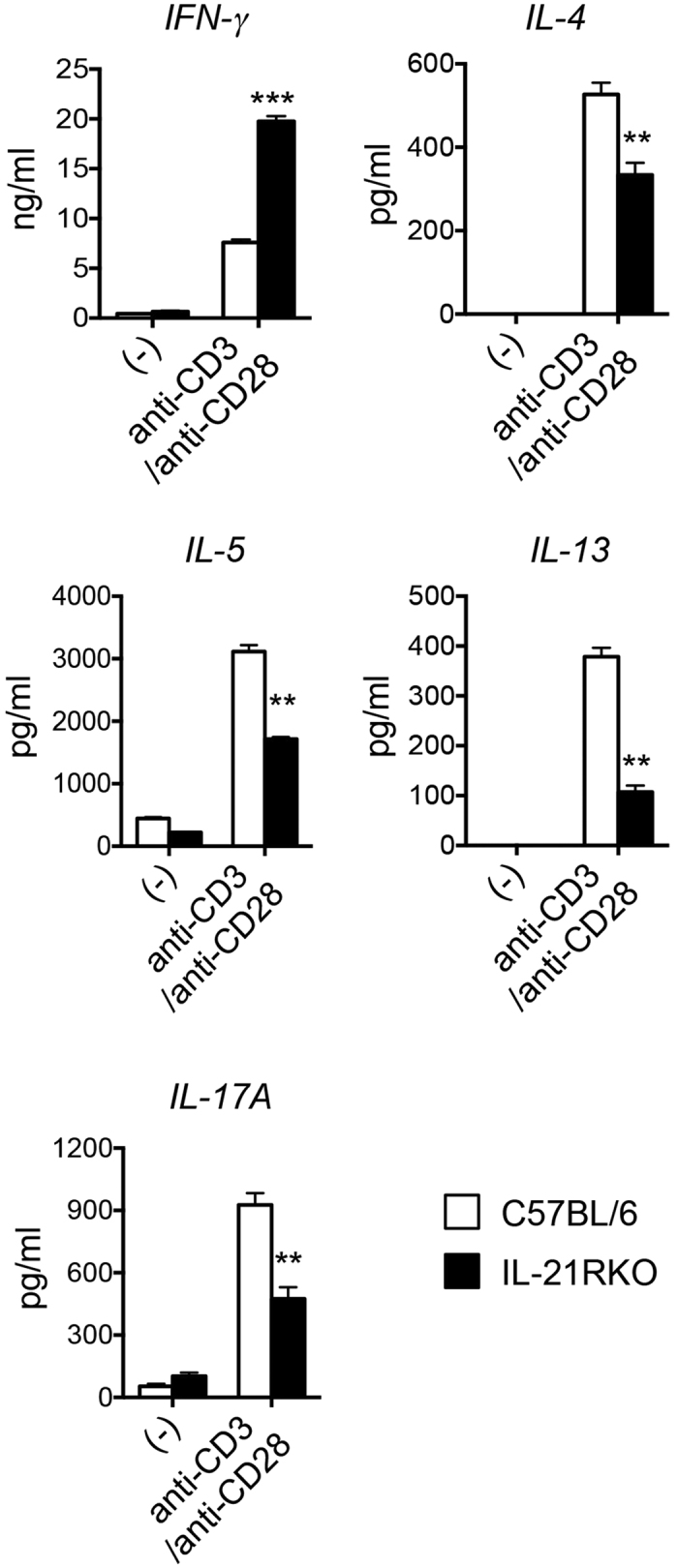
Th cell-associated cytokines productions in LP cells of colon after DSS-inducted colitis. LPL from colon of DSS-treated IL-21RKO or C57BL/6 mice was isolated and cultured for 48 hours with or without anti-CD3/anti-CD28 mAbs, and then cytokine production in the cultured supernatant was measured using ELISA kits. Data indicate mean ± SD of 5 mice of each group obtained from a representative of three independent experiments. Statistically significant differences are shown (**P < 0.01 or ***P < 0.001).

Next, we examined the level of cytokine production by LPL-T cells in intestine under the TCR stimulations on day 5 after DSS-administration. LPL was isolated and cultured with anti-CD3/anti-CD28 mAbs stimulations for 48 h, and then cytokine production in the culture supernatants were analyzed by ELISA. As shown in Fig. 5, the level of Th1 cytokine-IFN-γ was significantly higher, but the levels of Th2 cytokine-IL-4, IL-5 or IL-13, and Th17 cytokine-IL-17A were greatly lower in colonic LP of IL-21RKO mice after DSS-induced colitis as compared with those in C57BL/6 mice (**P < 0.01 or ***P < 0.001). All of these results fully demonstrate that Th1 response is enhanced, but Th2 and Th17 responses are inhibited in intestinal tissues of IL-21RKO mice during DSS-induced acute intestinal inflammation.

### Regulatory T Cell (Treg) Responses in LP during DSS-Induced Intestinal Inflammation

**Figure 6:**
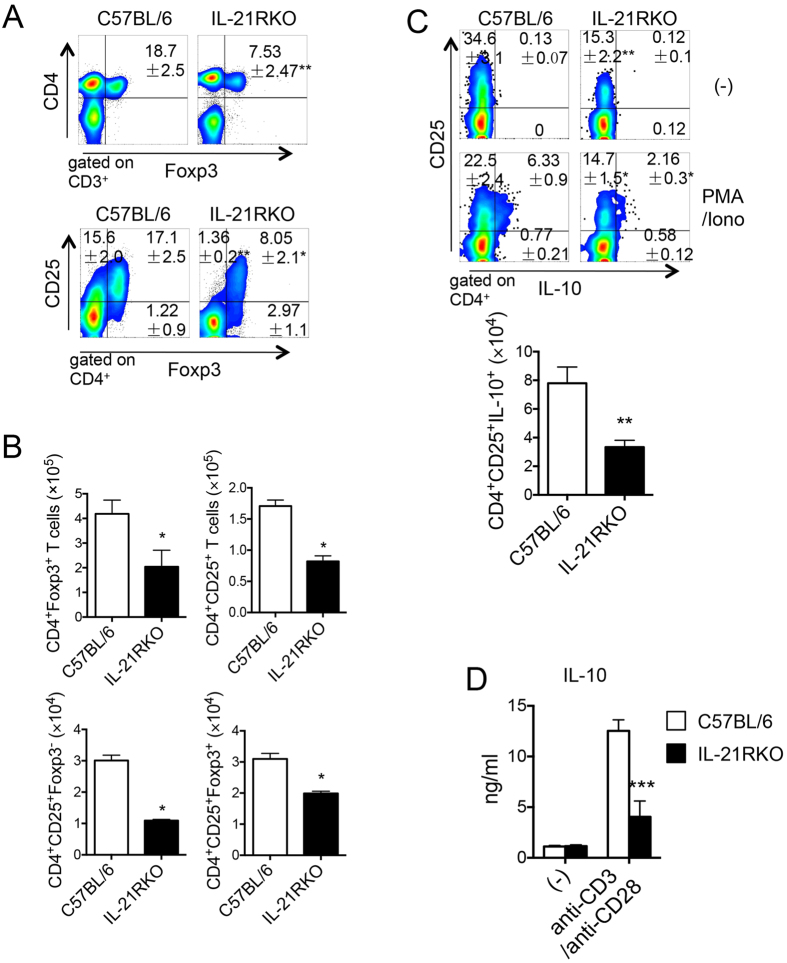
Effects of IL-21R deficiency on regulation of Treg responses in inflammatory intestine in mice. (**A**) The percentage of Foxp3^+^, CD25^+^, CD25^+^Foxp3^+^ or CD25^+^Foxp3^−^ in CD4^+^ T cell in LPL of mice with DSS-induced colitis; (**B**) The cell numbers of CD4^+^CD25^+^, CD4^+^Foxp3^+^, CD4^+^CD25^+^Foxp3^+^ or CD4^+^CD25^+^Foxp3^−^ cells in LPL; (**C**) The percentage and the absolute numbers of of CD4^+^CD25^+^IL-10^+^ T cells in LPL of colon. Data indicate mean ± SD of 3 mice of either group obtained from a representative of three independent experiments. (**D**) LPL was cultured for 48 h under the TCR stimulations, and then IL-10 production by T-LPL in the cultured supernatant was measured using ELISA kit. Data indicate mean ± SD of 5 mice of each group obtained from a representative of three independent experiments. Statistically significant differences are shown (*P < 0.05, **P < 0.01 or ***P < 0.001).

Treg cells could protect mice from DSS-induced intestinal inflammation through suppression the Th1 cell responses^32^. Finally, we investigated the effect of IL-21R deficiency on Treg cell responses in LP of colon after DSS-induced colitis. As compared with C57BL/6 mice, the expression of Foxp3 on CD4^+^ T cells significantly decreased in the IL-21RKO mice and the percentage of CD4^+^CD25^+^, CD4^+^CD25^+^Foxp3^+^ or CD4^+^CD25^+^Foxp3^−^ in CD4^+^ T cells was significantly lower (Fig. 6A, *P < 0.05), and the absolute numbers of these cell populations also markedly decreased in IL-21RKO mice (Fig. 6B, *P < 0.05). Moreover, both the expression of IL-10 in CD4^+^CD25^+^ T cells and the cell number of IL-10^+^CD25^+^CD4^+^ T cell significantly decreased in colonic LP of IL-21RKO mice as compared with those in C57BL/6 mice (Fig. 6C, *P < 0.05). The secretion of IL-10 protein by LPL-T cells in intestinal tissues of IL-21RKO mice evidently decreased as compared to C57BL/6 mice (Fig. 6D, ***P < 0.0001). These results evidence that IL-21/IL-21R signaling plays an important role in upregulation of Treg cell responses in inflammatory intestinal tissues in our experimental system.

### Stimulation of IL-21/IL-21R Signaling with rIL-21 significantly ameliorates DSS-induced intestinal inflammation in mice

**Figure 7:**
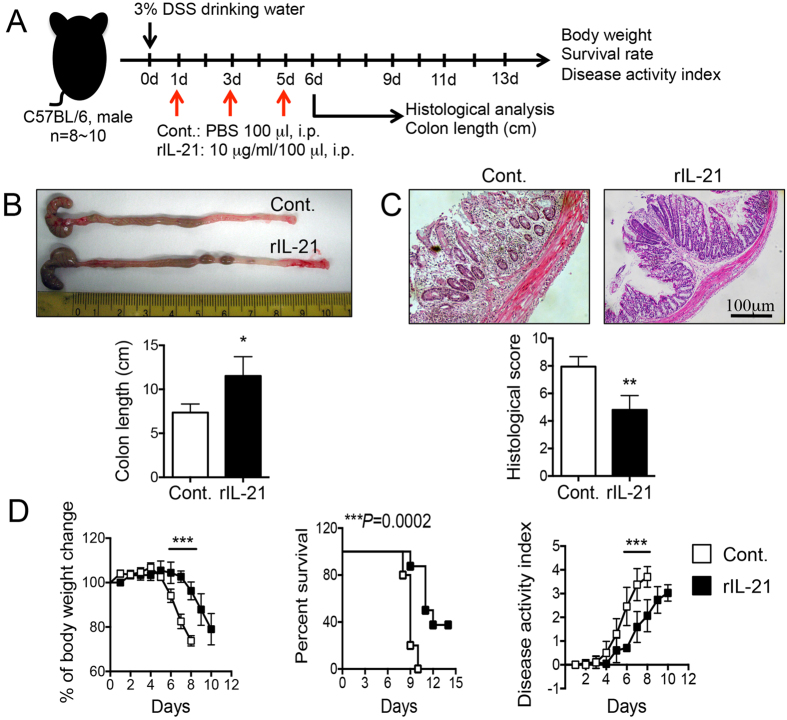
Effects of *in vivo* treatment with rIL-21 on DSS-induced colitis in mice. (**A**) The experimental design of *in vivo* treatment with rIL-21 on DSS-induced colitis in mice; (**B**) colon length (cm); (**C**) histological analysis; (**D**) body weight change, survival rate and DAI. Data indicate mean ± SD of 8~10 mice of each group obtained from a representative of two independent experiments. Statistically significant differences are shown (*P < 0.05, **P < 0.01 or ***P < 0.001).

**Figure 8:**
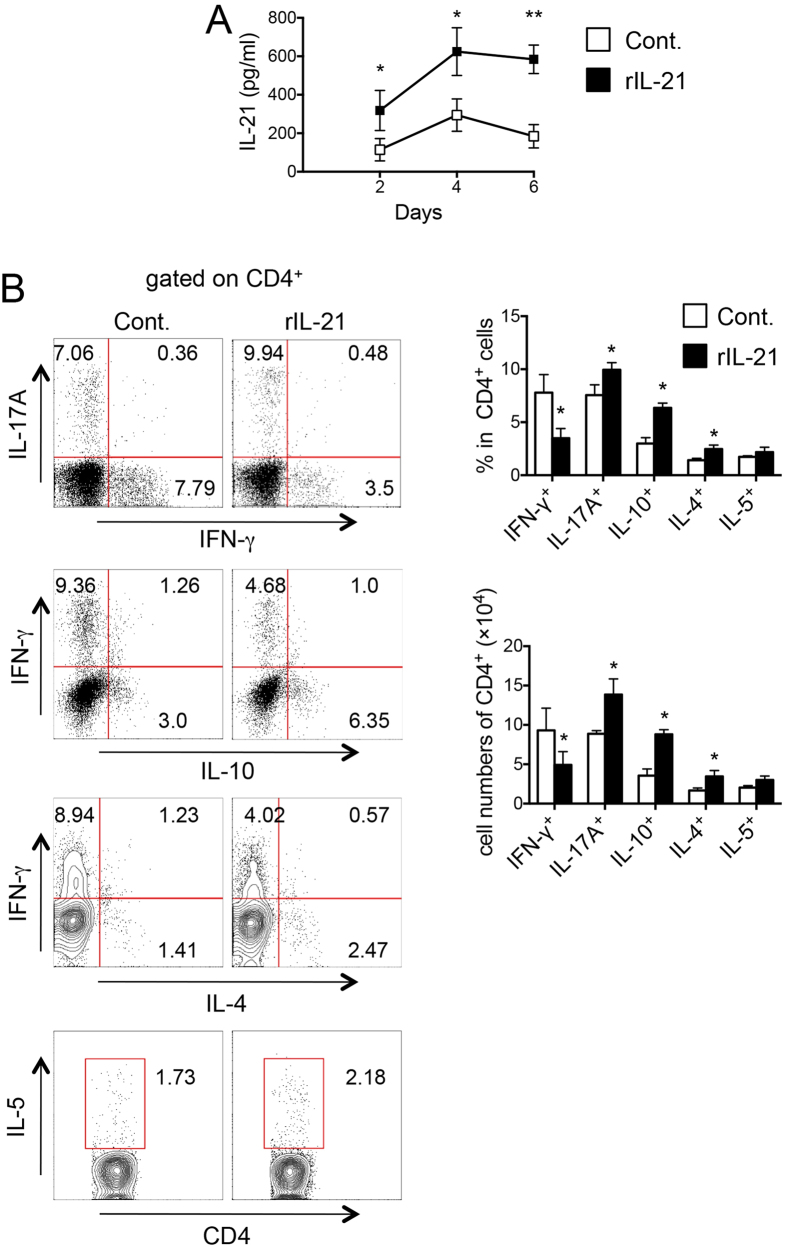
rIL-21-treatment improved intestinal inflammation induced by DSS in C57BL/6 mice. (**A**) IL-21 production by LP-T cell of colon from control or rIL-21-treated mice was analyzed on day 2, 4 and 6 after DSS-administration. (**B**) The percentage and absolute number of IFN-γ-, IL-17A-, IL-4- or IL-10-producing CD4^+^ LP-T cells from colon of control or rIL-21-treated mice were analyzed by cytokine FACS on day 6 after DSS-treatment. Data indicate mean ± SD of 3 mice of each group obtained from a representative of tow independent experiments. Statistically significant differences are shown (*P < 0.05 or **P < 0.01).

To elucidate the roles of IL-21/IL-21R signaling in DSS-induced colitis, we examined the effect of *in vivo* administration of rIL-21 on DSS-induced intestinal inflammation in C57BL/6 mice. Mice were injected with rIL-21 (100 μg/ml/100 μl) or PBS by i.p. on day 1, 3 and 5 after DSS-administration (Fig. 7A). *In vivo* treatment of rIL-21 significantly protected against intestinal inflammation induced by DSS in C57BL/6 mice as assessed by colon length, histological score, body weight change, survival rate and DAI (Fig. 7B–D, *P < 0.05, **P < 0.01 or ***P < 0.001). As shown in the Fig. 8A, the level of IL-21 secretion by LP-T cells from DSS-treated control mice after TCR stimulation (anti-CD3/anti-CD28) was increased gradually after DSS-administration, and peak appeared in day 4. Furthermore, the level of IL-21 production was significantly higher in colon of rIL-21-treated mice than those in control mice (*P < 0.05). The percentage and absolute number of IFN-γ-producing CD4^+^ LP-T cells were significantly reduced, but IL-17A-, IL-4- or IL-10-producing CD4^+^ LP-T cells were increased in rIL-21-treated mice as compared with those in control mice (Fig. 8B, *P < 0.05). These results suggest that IL-21/IL-21R signaling plays a protective role in the pathogenesis of DSS-induced acute colitis in mice mainly through regulation of Th cell responses in LP of colon. Therefore, rIL-21 may be useful as a novel biological therapy for CD.

## Discussion

Numberous studies have demonstrated that IBD-related inflammation is marked by elevated levels of IL-21, suggesting that this cytokine can play a key role in the detrimental response in these disorders^33^,^34^,^35^,^36^,^37^. However, a recent study showed that a homozygous mutation in IL-21 gene is associated with a common variable immunodeficiency-like B-cell deficiency that causes early-onset IBD in human subjects^38^. Moreover, Yeste *et al.* have recently shown that IL-21 induces IL-22 production in mucosal T cells, through a mechanism involving STAT3, ROR-γt and Aryl hydrocarbon receptor, thus protecting immune-deficient mice from DSS-induced colitis^39^. In line with this, our data have shown that IL-21/IL-21R signaling protects mice from DSS-induced colitis through regulation of Th cell-mediated immune responses in intestinal mucosa.

Genetic background is a key factor in determining the susceptibility of experimental rodents to DSS-induced colitis, as evident from the existence of marked species and strain differences^40^. The effector CD4^+^ T cells in C57BL/6 mice with DSS-induced colitis have long been considered as a Th1-type colitis animal model resembling CD^41^. Th1 cells inhibit the proliferation of Th2 cells which shut down IFN-γ production by Th1 cells, indicating that Th1 and Th2 cells are mutually regulated^42^,^43^. In this paper, we found that DSS-induced colitis was exacerbated in IL21RKO mice with C57BL/6 background, in which CD4^+^ T cells in the LP of large intestine produced higher levels of IFN-γ but less Th2-type cytokines than those in C57BL/6 mice. Thus, Th2-like immunity characterized by the productions of IL-4 and IL-5 was impaired, whereas Th1-like immunity capable of producing IFN-γ was enhanced in DSS-induced colitis model in IL-21RKO mice. These results proved that IL21/IL-21R signal played an important role in controlling intestinal inflammation by deviating the balance of Th1/Th2 to Th2 response in colon.

IL-21 has been thought to act as a “third signal” in CD8^+^ T cell activation through both a direct and indirect influence on CD8^+^ T cells^44^. We found that the amounts of CD8^+^ and CD8^+^CD44^+^ IFN-γ^+^ T-cells increased markedly in colonic LP of IL-21RKO mice with DSS-induced colitis. The finding was unexpected, given that IL-21 could cooperate with IL-7 or IL-15 to enhance proliferation and function of naive CD8^+^ T cells, and that IL-21 can promote the antitumor function of effector CD8^+^ T cells^45^. Nonetheless, it is consistent with reports that IL-21 can have the opposite effect from IL-2 on antigen-induced CD8^+^ T-cell differentiation. And that IL-21 antagonized IL-2-mediated induction of effector CD8^+^ T cells and counteracted the negative impact of IL-2 on ensuing antitumor efficacy^29^. However, the mechanism of suppressing CD8^+^ T cells by IL-21/IL21R signaling should be further studied.

Th17 cells feature prominently in inflamed tissues and are responsible for the protective and pathogenic inflammatory responses in autoimmune and infectious diseases^46^. IL-21 induced the Retinoic acid related orphan nuclear receptor-γ (RORγt), which is a transcription factor that functions as a master regulator of the Th17 cells^47^, and augmented expression of IL-23R on Th17 cells to promote the expansion of this cell population^48^. Studies in human beings, at least under specific inflammatory conditions, have been shown that IL-21 is made by cells producing preferentially IFN-γ^8^, the cytokine signature of Th1 cell responses, and not IL-17A^49^. In contrast, IL-21 is mainly produced by Th17 cell in mice^10^,^48^ and it plays a key role in the amplification of Th17 or Th2 cell responses^18^,^48^. In line with this, we found that Th17 (CD4^+^IL-17A^+^) and Th2 (CD4^+^IL-4^+^ and CD4^+^ IL-5^+^) obviously decreased in IL-21RKO mice with DSS-induced colitis.

Several studies have shown that IL-21 can inhibit the induction of Foxp3^+^ Tregs^10^,^47^. However, a recent study showed that Treg cells responses were upregulated in the DSS-induced colitis in mIL-21iso-Tg mice^50^. In this study, Treg cells significantly decreased in IL21RKO mice with DSS-induced colitis. The ability of IL-21 to inhibit the generation of Treg cells *in vivo* may be an indirect effect, resulting from IL-21-mediated inhibition of IL-2 production, which decreases the viability of Treg cells^25^. Because of deficiency of IL-21/IL-21R signal, there may be another inhibition factor plays a role in influence viability of Treg cells in IL-21RKO mice. IL-21 can also be immuno-suppressive because of its ability to induce IL-10 in naive CD4^+^ T cells, CD8^+^ T cells and B cells^51^. Cells that are polarized to either Th1 or Th17 populations in the presence of IL-21 also produce higher levels of IL-10^51^. IL-21 then induces the production of IL-10 by the immunosuppressive T-regulatory type1 (Tr1) cells, a subset of regulatory cells, which lack Foxp3 expression and produce IL-10^52^. In this study, CD4^+^CD25^+^Foxp3^−^ cells and the production of IL-10 significantly decreased in IL-21RKO mice with DSS-induced colitis, which may contribute to aggravate the development of colitis. The reason may be the deletion of IL-21/IL-21R signaling.

Therefore, the high complexity of human IBD and the conflicting roles of IL-21/IL21R signaling in different immune cells make it hard to define IL-21/IL-21R signaling as a therapeutic target. However, it should be considered that the physiological effects of IL-21/IL-21R comprise more than its role as an amplifier or alleviative of inflammation. We should keep in mind that a fine balance of pro- and anti-inflammatory factors is necessary to preserve intestinal homeostasis. In IBD, this balance is broken and under certain conditions, the intervention with immune-shifting factor like IL-21/IL-21R signaling might be helpful to counterbalance excessive T cell-mediated inflammatory reaction.

## Materials and Methods

### Mice

The generation of IL-21RKO mice with C57BL/6 background was described previously^53^. IL-21RKO mice with C57BL/6 background were backcrossed into C57BL/6 strain more than eight times, and their littermates were used as control. This study was approved by the Committee of Ethics on Animal Experiment in Faculty of Medicine, China Medical University. Experiments were carried out under the control of the Guidelines for Animal Experiments.

### Abs and reagents

Abs for FACS analysis, Fcγ receptor-blocking mAb (CD16/32; 2.4G2), FITC-conjugated anti-γδTCR (GL-3), anti-CD4 (RM4-5), anti-CD8α (53-6.7), anti-CD25 (3C7), anti-Foxp3 (MF23), anti-IL-10 (JES5-16E3), anti-IL-17A (TC11-18H10.1), anti-IL-4 (11B11) and anti-IL-2 (JES6-5H4); APC-conjugated anti-CD44 (IM7) and anti-CD3ε (145-2C11); PE-conjugated anti-CD8α (53-6.7), anti-CD25 (3C7), anti-NK1.1 (PK136), anti-IFN-γ (XMG1.2), anti-IL-10 (JES5-16E3) and anti-IL-5 (TRFK5); PerCP-conjugated anti-CD4 (RM4-5) and anti-CD8α (53-6.7) mAbs were purchased from Biolegend (San Diego, CA). Purified anti-CD3 and anti-CD28 (37.51) mAbs were obtained from BD Biosciences, (San Diego, CA).

### Induction of acute colitis by DSS

For acute colitis induction by DSS, mice were administered 3% (weight/volume) DSS (molecular weight 36–50 kilodaltons; ICN Biomedicals, Aurora, Ohio) in their drinking water. The body weight, survival rate and disease activity index (DAI) of each animal were monitored daily. DAI was determined by scoring the loss of body weight, trait of stool, and occult blood in stool or hematochezia according to the classic scoring system by Cooper^54^. The scoring process is given as follow: body weight loss (0, none; 1, 1–5%; 2, 5–10%; 3, 10–20%; 4, >20%), stool consistency (0, normal; 2, loose stool; 4, diarrhea), and stool blood (0, negative; 2, fecal occult blood test positive; 4, gross bleeding). Mice were sacrificed on day 5 or 6 in acute colitis for measure colon length, and the tissues of colons were removed and cleaned. Sections were taken for cell culture, flow cytometry and histology.

### Histological assessment of colitis

The middle part of the colon was fixed with 4% paraformaldehyde, and the fixed tissue was embedded in paraffin. Five-micrometer tissue sections were sliced and stained with ematoxylin-eosin stain. Histology was scored as described previously^55^: epithelium (E): 0, normal morphology; 1, loss of globlet cells; 2, loss of globlet cells in large areas; 3, loss of crypts; 4, loss of crypts in large areas; and infiltration (I): 0, no infiltrate; 1, infiltrate around the crypt basis; 2, infiltrate reaching the L muscularis mucosae; 3, extensive infiltration reaching the L muscularis mucosae and thickening of the mucosa with abundant edema; 4, infiltration of the L submucosa. The total histologic score was given as E + I.

### Lamina Propria Lymphocytes (LPL) Preparation

LPL in the colon were isolated as described previously^55^. In brief, section of the colon tissue was cut into 2-mm slices and the epithelium was eliminated by stirring, first in PBS containing 3 mM EDTA for 10 min at 37 °C (twice) and then in RPMI 1640 (Thermo Fisher Scientific, MA, USA) containing1% FBS (Thermo Fisher Scientific, MA, USA), 1 mM EGTA, and 1.5 mM MgCl_2_ for 15 min (also twice). The samples were collected and further stirred in RPMI containing 20% FBS, 100 U/mL collagenase (Sigma-Aldrich Corp., St. Louis, MO, USA), and 5 U/mL DNase 1 (Sigma-Aldrich Corp. St. Louis, MO, USA) for 90 min at 37 °C. Halfway through the incubation and at the end of the incubation, the suspension was subjected to multiple aspirations performed by passing it through a syringe for 40–50 times followed by differential centrifugation in a 45%/66.6% discontinuous Percoll (Solarbio, Beijing, China) gradient at 2500 rpm for 20 min. The number of viable LPL was counted after staining with trypan blue.

### Flow Cytometry Analysis

For flow cytometry analysis, isolated LPL were preincubated with an Fc receptorblocking mAb (CD16/32; 2.4G2) for 15 minutes at 4 °C then incubated with saturating amounts of FITC-, PE-, APC-, and PerCP-conjugated mAbs for 30 minutes at 4 °C. For intracellular cytokine staining, LPL were stimulated with PMA (25 ng/ml; SigmaAldrich, St. Louis, MO) and ionomycin (1 μg/ml; Sigma-Aldrich) for 5 h at 37 °C. Brefeldin A (10 μg/ml; Sigma-Aldrich) was added after the first hour of incubation. These cells were harvested, washed, and stained with mAbs for surface staining then cells were subjected to intracellular cytokine staining using a Cytofix/Cytoperm Kit Plus (BD Biosciences, San Jose, CA) according to the manufacturer’s instructions and the fluorescence of the cells was analyzed using BD LSRFortessa (BD, San Jose, CA). The data were analyzed with FlowJo software (TreeStar, San Carlos, CA).

### Cytokine analysis by Enzyme-Linked Immunosorbent Assay

To measure spontaneous cytokine production by LPL, LPL were cultured in 96-well flat-bottom plate (Falcon; BD Biosciences) without any stimulation for 24 h at 37 °C under 5% CO_2_. To measure cytokine production by LPL-T cell, isolated LPL was incubated for 48 h *in vitro* in 96-well flat-bottom plate coating with anti-CD3ε (10 μg/ml) and soluble anti-CD28 (1 μg/ml) antibodies (BD Biosciences, San Diego, CA). The culture supernatants were then harvested and assayed for cytokine concentration with ELISA kits (R&D Systems) according to the manufacturer’s instruction.

### *In vivo* Recombinat mouse IL-21-Treatment

C57BL/6 mice were treated with 3% DSS in drinking water, and intraperitoneal injected with recombinat mouse IL-21 (rIL-21, carrier free, Biolegend, San Diego, CA), 10 μg/ml/100 μl on day 1, 3 and 5 after DSS-treatment. The body weight change, survival rate and DAI were observed daily. Histological analysis and colon length (cm) were measured on day 6 after DSS-induced colitis.

### IL-21 production analysis by Enzyme-Linked Immunosorbent Assay

To measure IL-21 production by LPL-T cell, LPL of colon from control or rIL-21-treated mice were isolated on day 2, 4 or 6 after DSS-treatment and incubated for 48 h *in vitro* in 96-well flat-bottom plate coating with anti-CD3ε (10 μg/ml) and soluble anti-CD28 (1 μg/ml) antibodies (BD Biosciences, San Diego, CA). The culture supernatants were then harvested and assayed for IL-21 concentration with ELISA kits (R&D Systems) according to the manufacturer’s instruction.

### Statistical Analysis

The difference in survival rates was assessed by the log rank test (Mantel-Cox). Disease activity index and histologic scores were statistically analyzed using the Mann–Whitney U test. Differences in parametric data were evaluated by a Student’s *t* test. Statistically significant differences were accepted at the *p* < 0.05.

## Additional Information

**How to cite this article**: Wang, Y. *et al.* IL-21/IL-21R signaling suppresses intestinal inflammation induced by DSS through regulation of Th responses in lamina propria in mice. *Sci. Rep.* **6**, 31881; doi: 10.1038/srep31881 (2016).
